# Thermal Deformation Modeling for Phased Array Antenna Compensation Control

**DOI:** 10.3390/s22062325

**Published:** 2022-03-17

**Authors:** Hui Liu, Wei Wang, Dafeng Tang, Liyin Zhang, Youming Wang, Enming Miao

**Affiliations:** 1 School of Automation, Xi’an University of Posts & Telecommunications, Xi’an 710121, China; liuhui@xupt.edu.cn (H.L.); tdflyy2019@xupt.edu.cn (D.T.); zhangliyin@xupt.edu.cn (L.Z.); wangyouming@xupt.edu.cn (Y.W.); 2 School of Mechano-Electronic Engineering, Xidian University, Xi’an 710071, China; wwang@xidian.edu.cn; 3 College of Mechanical Engineering, Chongqing University of Technology, Chongqing 400054, China

**Keywords:** thermal deformation, finite element analysis, data-driven modeling, error compensation, phased array antenna

## Abstract

Thermal compensation control can correct errors caused by the thermal deformation of phased array antenna (PAA) panels. Thermal deformation of the panel is needed to calculate the compensation value. While the PAA is working, thermal deformation is unconditional to measure, but predicting it by temperature is feasible. However, thermal deformation is also affected by other factors, such as the structural shape, assembly method, and material parameters, and it is difficult to measure these parameters of PAA because of the complex structure. In contrast, the measurement method of the temperature and thermal deformation of the PAA in the laboratory is much easier. Therefore, a comprehensive influence parameters (CIPs)-finite element method (FEM) method was proposed in this study, it can extract the influence of above parameters on thermal deformation from temperature and thermal deformation measurement data and build a thermal deformation prediction model. Experiments have verified that the CIPs-FEM can greatly reduce the difficulty of thermal deformation modeling and have a high prediction accuracy.

## 1. Introduction

The phased array antenna (PAA) is an array of multiple T/R modules. The emission direction of the electromagnetic wave depends on the phase differences of all T/R modules [1]. The power of each T/R module is in the KW level [2], so, the T/R module generates a lot of heat. Therefore, the panel of the PAA has a sizeable thermal deformation. The phase of the electromagnetic wave emitted by the T/R module is divided into the in-array phase and the space phase. The in-array phase is controlled by the phased shifter of the T/R module, and the space phase depends on the position of the T/R module on the panel. The thermal deformation changes the space phase and cause a decay in the electrical performance [3]. The thermal deformation problem of the PAA was noticed very early. Li [4] studied the thermal deformation of a spaceborne antenna in 1984 and proposed a method to measure thermal deformation based on the changes in the electrical signal of the antenna. However, the measurement principle depends on the structure of the antenna and is not universal. Wang [5] found that thermal deformation causes the gain of the main lobe to decay. Wang et al. [2] and Wang et al. [6] established a coupled structural–electromagnetic–thermal model by the finite element method (FEM), which provides some theoretical guidances for the structure design of the phased array antenna. H. Ren [7], Han et al. [8], and Zhang et al. [9] optimized and simulated the heat dissipation system of a phased array radar. However, the simulation results show that the antenna still has a significant temperature rise, and the heat dissipation system can only ensure the regular operation of the electronic chip of the antenna. Thus, the thermal deformation is still a difficult problem for the PAA to solve.

The electrical performance decay of the PAA caused by the phase changes can be corrected by the compensation control method [10,11,12]. Theoretically, if the thermal deformation is known, the change of the space phase $\left({∆}_{\varphi s}\right)$ can be calculated, and the electrical performance can be compensated by controlling the in-array phase (the compensation value is $-{∆}_{\varphi s}$ ) [13,14]. In the laboratory, some optical methods are suitable for measuring thermal deformation [15,16]. Lebron developed a dedicated equipment that can measure thermal deformation and temperature simultaneously [17]. However, while the PAA is working, these methods cannot be used, because there is no place to install measurement devices for many PAAs such as airborne radars. Therefore, how to obtain thermal deformation is the biggest problem.

Currently, the temperature is used to predict the thermal deformation of mechanical equipment in many studies (thermal deformation is a common problem of many mechanical devices). Thermal deformation is also affected by other factors such as the structural shape, assembly method, and material parameters (referred to as thermal deformation influence parameters in the following text) [5]. For example, Xu [18] and Hayashi et al. [19] found the location of the bolt and differences in the thermal expansion coefficients of different materials affect the thermal deformation. It is not easy to accurately measure the thermal deformation influence parameters. Then, the current studies can only predict the thermal deformation of some simple structures, such as ball screws [20,21] and spindles [22]. In addition, some necessary simplifications and assumptions have to be made for prediction, but this will also cause a decrease in the accuracy. The panel of the PAA is very complex, and there are many connections and fixing devices for assembly and installation. Therefore, it is too difficult to measure the influence parameters for predicting thermal deformation, and no relevant research has been conducted so far.

Theoretically, the influence of the structural shape, assembly method, and material parameters on thermal deformation is implicit in the real thermal deformation, and it is easy to directly measure the thermal deformation and temperature of the PAA panel in the laboratory [15,16,17]. Therefore, can the influence of thermal deformation influence parameters be extracted from thermal deformation and temperature measurement data and used for prediction? Based on this, a new thermal deformation modeling method combined with the FEM and a data-driven model was proposed in this study, in which comprehensive influence parameters, namely CIPs, were introduced in the FEM. The influence of thermal deformation influence parameters on thermal deformation can be converted into the CIPs. Based on the measurement data of temperature and thermal deformation, the inverse finite element modeling (IFEM) algorithm was proposed to calculate the CIPs. Then, the temperature and thermal deformation were needed for modeling, instead of the thermal deformation influence parameters. The proposed method is referred to as CIPs-FEM, and it can reduce the difficulty of thermal deformation modeling and has a high prediction accuracy.

## 2. Thermal Deformation Modeling Method

The constitutive equations between the temperature and the thermal stress are shown as Equations (1)–(3):

(1) $$\varepsilon_{T}=\left[\varepsilon_{xT}\;\varepsilon_{yT}\;\varepsilon_{zT}\;0\;0\;0\right],\varepsilon_{xT}=\varepsilon_{yT}=\varepsilon_{zT}=\alpha ∆T,$$

(2) $$\sigma_{T}=D\varepsilon_{T},$$

(3) $$D=\frac{E\left(1-v\right)}{\left(1+v\right)\left(1-2v\right)}\left[\begin{array}{cccccc} 1 & \frac{v}{1-v} & \frac{v}{1-v} & 0 & 0 & 0\\ \frac{v}{1-v} & 1 & \frac{v}{1-v} & 0 & 0 & 0\\ \frac{v}{1-v} & \frac{v}{1-v} & 1 & 0 & 0 & 0\\ 0 & 0 & 0 & \frac{1-2v}{2\left(1-v\right)} & 0 & 0\\ 0 & 0 & 0 & 0 & \frac{1-2v}{2\left(1-v\right)} & 0\\ 0 & 0 & 0 & 0 & 0 & \frac{1-2v}{2\left(1-v\right)} \end{array}\right],$$

where $\varepsilon_{T}$ is the thermal strain, $\sigma_{T}$ is the thermal stress, D is the elastic matrix, E is the elastic modulus, v is Poisson’s ratio, and α is the thermal expansion coefficient.

Due to the influence of the thermal deformation influence parameters, the coefficient of thermal expansion of the panel was not uniform. Therefore, CIPs are introduced in Equation (1), as shown in Equation (4):

(4) $$\varepsilon_{T}=\left[\varepsilon_{xT}\;\varepsilon_{yT}\;\varepsilon_{zT}\;\gamma_{yzT}\;\gamma_{xzT}\;\;\gamma_{xyT}\;\right]=\;\left[\begin{array}{ccc} \Omega_{T,x}^{e} & \Omega_{T,y}^{e} & \Omega_{T,z}^{e} \end{array}\begin{array}{ccc} \;\;\Omega_{T,yz}^{e} & \Omega_{T,xz}^{e} & \Omega_{T,xy}^{e} \end{array}\right]\cdot \alpha ∆T=\Omega_{T}^{e}\alpha ∆T$$

where $\varepsilon_{xT},\;\varepsilon_{yT},\;\mathrm{and}\;\varepsilon_{zT}$ are the thermal normal strains in the X, Y, and Z directions, respectively, $\gamma_{yzT}\;\gamma_{xzT}\;\gamma_{xyT}$ are the thermal shearing strains in the X, Y, and Z directions, respectively, and $\begin{array}{ccc} \Omega_{T,x}^{e} & \Omega_{T,y}^{e} & \Omega_{T,z}^{e} \end{array}\begin{array}{ccc} \;\Omega_{T,yz}^{e} & \Omega_{T,xz}^{e} & \Omega_{T,xy}^{e} \end{array}$ are CIPs. To fit the difference of the coefficient of thermal expansion, the CIPs at different positions of the panel are different.

Then, the panel is divided into elements. The CIPs in Equation (4) are independently introduced into each element, and the element stiffness equation is established by the principle of the minimum potential energy [23], as shown in Equation (5):

(5) $$K^{e}\delta^{e}=f_{T}^{e},$$

where $K^{e}$ is the element stiffness matrix and described as:

(6) $$K^{e}=\overset{\;}{\underset{V}{∭}}N^{T}JDJ^{T}NdV\;\;\;\;$$

$\delta^{e}$ and $f_{T}^{e}$ are the nodal thermal deformation and the equivalent nodal force of nodes of one element, respectively, and $f_{T}^{e}$ is expressed as:

(7) $$f_{T}^{e}={∭}_{V}\;N^{T}JD\Omega_{T}^{e}{}^{T}\alpha ∆TdV,$$

where ${*}^{T}$ means the transpose, N is the shape function matrix and is determined after the element is divided, and J is the differential operator and described as:

(8) $$\left[\begin{array}{ccc} \frac{\partial }{\partial x} & 0 & 0\\ 0 & \frac{\partial }{\partial y} & 0\\ 0 & 0 & \frac{\partial }{\partial z}\; \end{array}\;\begin{array}{ccc} 0 & \frac{\partial }{\partial z} & \frac{\partial }{\partial y}\\ \frac{\partial }{\partial z} & 0 & \frac{\partial }{\partial x}\\ \frac{\partial }{\partial y} & \frac{\partial }{\partial x} & 0 \end{array}\right].$$

The overall stiffness equation is synthesized, as the displacements of the coincident nodes of adjacent elements are equal, as shown in Equation (9):

(9) $$K\delta =f_{T},$$

where K is the overall stiffness matrix, δ and $f_{T}$ are the nodal thermal deformations and equivalent nodal forces of all nodes. The thermal deformation can be calculated by solving Equation (9) with the FEM. This modeling method is referred to as the CIPs-FEM.

**The solution method of CIPs is as follows.**

The IFME algorithm was proposed to obtain the CIPs according to the measurement data of temperature and thermal deformation in this study. 

The CIPs are set to the unknown in Equation (9). After introducing the thermal deformation and temperature measurement data, the δ and the ∆T in Equation (9) are known. Therefore, the remaining unknown parameters in Equation (9) are only the CIPs. Then, the CIPs of each element can be solved by the least squares algorithm, as shown in Equation (10):

(10) $$\Gamma_{S}=\sum {\left(\delta -K^{-1}f_{T}\right)}^{2}\to min.$$

The specific calculation method is as follows:

(11) $$\{\begin{array}{c} \frac{\partial \Gamma_{S}}{\partial \Omega_{T,x}^{e_{1}}}=0\\ \vdots \\ \frac{\partial \Gamma_{S}}{\partial \Omega_{T,z}^{e_{\Lambda }}}=0 \end{array}\Rightarrow \left(\begin{array}{ccc} A_{1,1} & \cdots & A_{1,3\Lambda }\\ \vdots & \ddots & \vdots \\ A_{3\Lambda ,1} & \cdots & A_{3\Lambda ,3\Lambda } \end{array}\right)\Omega_{T}=\left(\begin{array}{c} A_{1,0}\\ \vdots \\ A_{3\Lambda ,0} \end{array}\right),$$

(12) $$\Omega_{T}={\left(\begin{array}{ccc} \begin{array}{ccc} \Omega_{T,x}^{e_{1}} & \Omega_{T,y}^{e_{1}} & \Omega_{T,z}^{e_{1}} \end{array} & \cdots & \begin{array}{ccc} \Omega_{T,yz}^{e_{\Lambda }} & \Omega_{T,xz}^{e_{\Lambda }} & \Omega_{T,xy}^{e_{\Lambda }} \end{array} \end{array}\right)}^{T},$$

where $\Omega_{T}$ is a vector consisting of all CIPs, $A_{1,1,\ldots ,}A_{3\Lambda ,3\Lambda }$ are the coefficients of $\Omega_{T}$, and $A_{1,0},\ldots ,A_{3\Lambda ,0}$ are constant terms. Finally, the $\Omega_{T}$ (CIPs) can be obtained by solving Equation (11), as follows:

(13) $$\Omega_{T}={\left(\begin{array}{ccc} A_{1,1} & \cdots & A_{1,3\Lambda }\\ \vdots & \ddots & \vdots \\ A_{3\Lambda ,1} & \cdots & A_{3\Lambda ,3\Lambda } \end{array}\right)}^{-1}\left(\begin{array}{c} A_{1,0}\\ \vdots \\ A_{3\Lambda ,0} \end{array}\right).$$

The values of $A_{1,0,\ldots ,}A_{3\Lambda ,3\Lambda }$ can be calculated by the symbolic variables in the derivative function of Matlab:

1. The elements in $\Omega_{T}$ are set as symbol variables (Matlab code: syms… real);
2. Symbolic variables are used to calculate $\Gamma_{S}$ in Equation (10);
3. The function of derivation twice (Matlab code: diff) is used to calculate any value in $A_{1,1,\ldots ,}A_{3\Lambda ,3\Lambda }$.
  For example, $A_{1,1}$ is the coefficient of $\Omega_{T,x}^{e_{1}}$ in the $\frac{\partial \Gamma_{S}}{\partial \Omega_{T,x}^{e_{1}}}=0$. The calculation method is shown as Equation (14):
  (14) $$A_{1,1}=\frac{\partial \frac{\partial \Gamma_{S}}{\partial \Omega_{T,x}^{e_{1}}}}{\partial \Omega_{T,x}^{e_{1}}}.$$
4. After finding all the coefficients in an equation, the constant term can be found.
  For example, $A_{1,0}$ is the constant term of the $\frac{\partial \Gamma_{S}}{\partial \Omega_{T,x}^{e_{1}}}=0$. The calculation method is as Equation (15):
  (15) $$A_{1,0}=\frac{\partial \Gamma_{S}}{\partial \Omega_{T,x}^{e_{1}}}-\left(\begin{array}{ccc} A_{1,1} & \cdots & A_{1,3\Lambda } \end{array}\right)\Omega_{T}.$$
5. After the calculation is completed, the symbolic variable can be converted into an ordinary variable (Matlab code: eval).

## 3. Experiment of Thermal Deformation Modeling

A simulation panel that can simulate the heating process of the PAA is made. Based on the temperature and thermal deformation measurement data of the panel, the thermal deformation modeling effects of the CIPs-FEM and the traditional thermal deformation calculation method (FEM) were compared.

### 3.1. Experiment Devices

**Figure 1:**
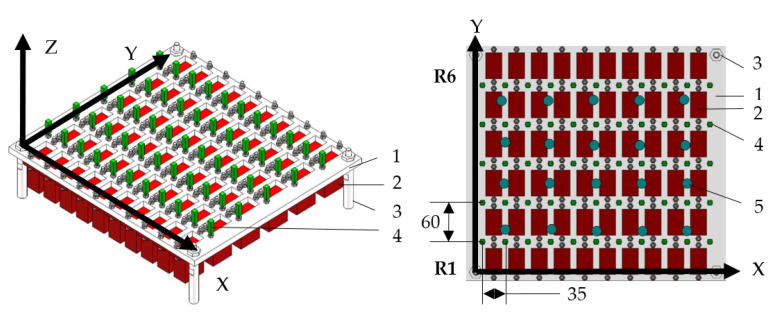
Schematic diagram of the simulation panel. 1. panel; 2. heater (10×6); 3. column (2×2); 4. cuboid measuring standard part (11×5); 5. temperature sensor (number: 20).

**Figure 2:**
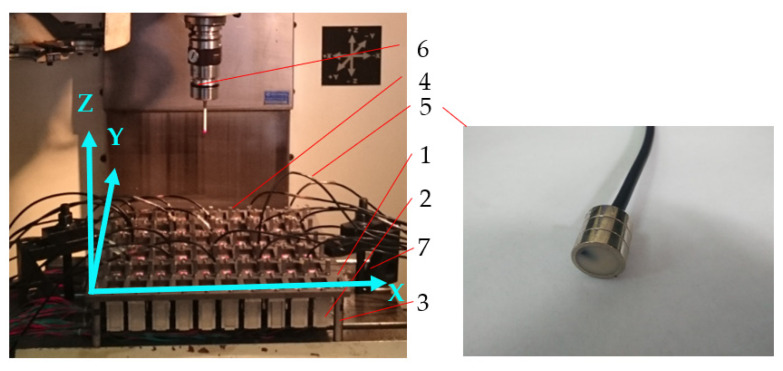
Photo of experimental devices. 1. panel; 2. heater (10×6); 3. column (2×2); 4. cuboid measuring standard part (11×5); 5. temperature sensor (number: 20); 6. probe of a coordinate-measuring machine (CMM); 7. fixed devices (number: 2).

As shown in Figure 1 and Figure 2, the simulation panel made of 45# steel was rectangular, the size in the X, Y, and Z axes were 400, 360, and 10 mm, respectively. The six rows (R1–R6) of the heater were installed under the panel to simulate the heating of T/R modules. There were 10 heaters in each row, and each row had a power supply device, which could adjust the voltage independently. Above the panel, 55 (calculated as 11 × 5) thermal deformation measurement points were evenly arranged, and a cuboid measuring standard part was installed at each thermal deformation measurement point. Then, the thermal deformations in the X, Y, and Z axes of each point can be measured by a coordinate-measuring machine (CMM). The panel was placed on the worktable of the CMM and fixed by clamping. Twenty temperature sensors were evenly arranged on the panel to measure the temperature, to prevent the measurement of thermal deformation from being interfered by the transmission line of the temperature sensors. The temperature sensor model was DS18B20, which was encapsulated in a magnetic adsorption probe with a thermal grease. It could be installed and disassembled quickly when needed.

### 3.2. Experiment Process

A total of 9 batches of experiments were performed in this study, referred to as M1 and P1–P8. M1 was used for modeling the deformation, and P1–P8 were used to test the prediction effect of the model. The processes of all experiments were the same, but the voltages of the heaters were different (shown in Table 1). The details of the experiment process were as follows:

1. All the temperature sensors were placed on the panel, and the initial temperature was recorded. Then, all temperature sensors were removed, and the initial positions of all thermal deformation measurement points in the X, Y, and Z axes were measured;
2. All the temperature sensors were placed on the panel, and the heaters were turned on. After it was stabilized, the temperature was recorded. Then, all temperature sensors were removed, and the positions of all thermal deformation measurement points in the X, Y, and Z axes were measured. The temperature rise and the thermal deformation were calculated as: the measured value in step (2) minus the initial value in step (1).

### 3.3. Experiment Results

**Figure 3:**
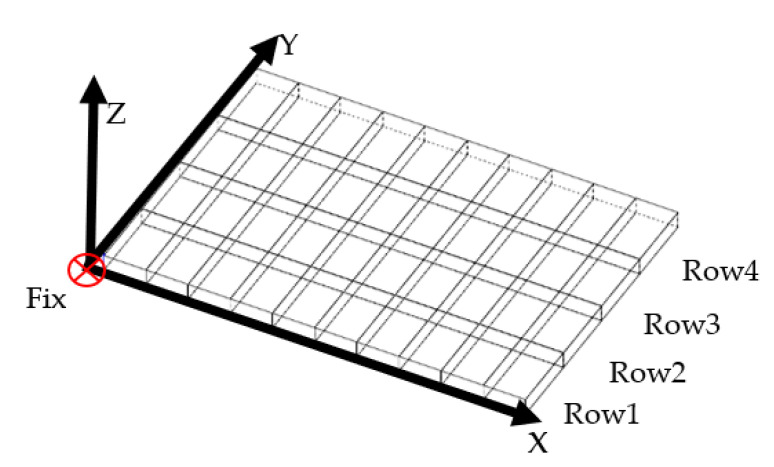
Element divisions of the simulation panel.

As shown in Figure 3, the structure of the panel was simplified for modeling. Then, the modeling effect of the CIPs-FEM can be verified on the simplified panel. Each thermal deformation measurement point was taken as a node, and the panel was divided into cuboid elements. A node was fixed as a constraint, and the thermal deformation of this node was set to 0. Although the panel was measured only on one side, the panel was thinner, so the thermal deformations and temperatures on both sides were considered the same. The relevant material parameters involved in the calculation were as follows:

Elastic modulus *E*: 206 GPa;

Poisson’s ratio *v*: 0.3;

Thermal expansion coefficient α: $11.59\times 10^{-6}$/°C.

The data of M1 were used for modeling. Because the node was the deformation measurement point, the thermal deformation data were directly brought into the divided elements. For the temperature data, according to Figure 3, the direction of rows of elements in the X axis was parallel to the direction of rows of heaters. The temperatures of elements in the same row were equal because the supply voltage of heaters in the same row is equal. Therefore, the mean value of five temperature sensors placed in one elements row was taken as the temperature of elements in this row. The temperature measurement results are shown in Table 2.

The temperature data of P1–P8 were taken into the CIPs-FEM and traditional FEM separately to predict thermal deformation. Then, the residual standard deviation (*RSD*) was used to measure the prediction accuracy, in Equation (16) [24]:

(16) $$RSD=\sqrt{\frac{\sum_{i=1}^{N}{\left(d_{i}-{\hat{d}}_{i}\right)}^{2}}{N-1}}\;,$$

where $d_{i}$ is the deformation measurement value of node i, $\;{\hat{d}}_{i}$ is the corresponding prediction value, and N is the number of nodes. The smaller the *RSD*, the higher the prediction accuracy.

**Figure 4:**
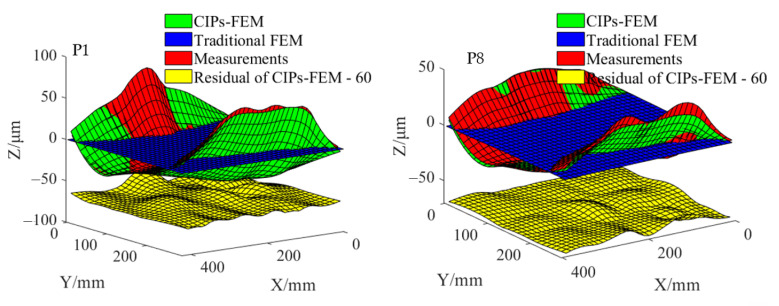
Thermal deformation prediction results.

**Figure 5:**
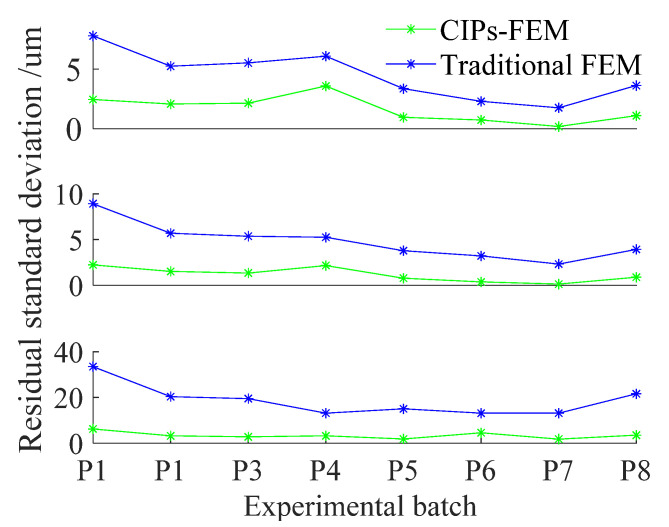
Thermal deformation prediction residual standard deviations (*RSDs*) of P1–P8.

Figure 4 shows the deformation prediction results of P1 and P8. For ease of observation, the thermal deformations in the X, Y, and Z axes were magnified 1000 times, and the residuals in Figure 4 were subtracted by 60. The *RSDs* of P1–P8 are shown in Figure 5.

In Figure 5, the *RSD* of the CIPs-FEM was smaller than that of the FEM. The mean *RSDs* of P1–P8 in the X, Y, and Z axes were 0.8, 0.9, and 2.0 μm, respectively, for the CIPs-FEM and 4.0, 6.0, and 26.4 μm, respectively, for the FEM. The increases in the accuracy were up to 80%, 85%, and 92%, 80%, with an average value of 85.6%.

## 4. Test of Thermal Compensation Control of the PAA

Based on the thermal deformation prediction data, the performance compensation control effect of the PAA was tested by simulation. 

### 4.1. Principle of the Thermal Compensation Control of the PAA

**Figure 6:**
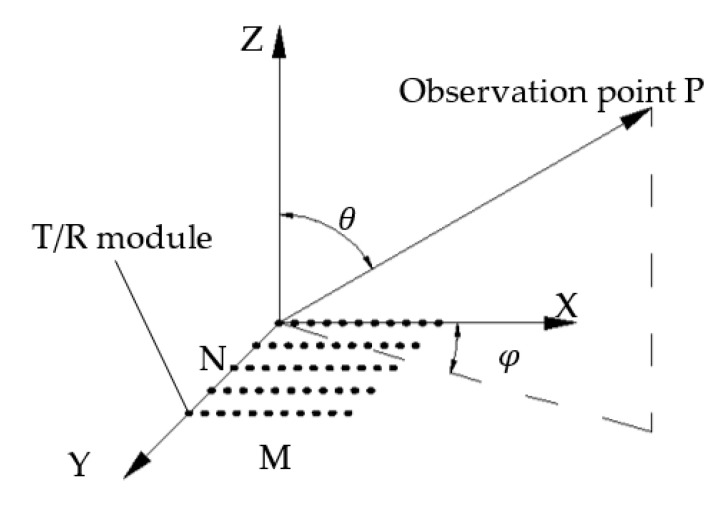
Schematic diagram of a rectangular array of the phased array antenna (PAA).

Figure 6 is a schematic diagram of a rectangular array of the PAA. The normalized far-field pattern of observation point P depends on the phase difference of T/R modules, as shown in Equation (17):

(17) $$\left(\varphi ,\theta \right)=10log\left(\frac{4\pi {\left(\sum_{m=1}^{M}\sum_{n=1}^{N}e^{j\left(\varphi_{I}{}_{m,n}+\varphi_{S}{}_{m,n}\right)}\right)}^{2}}{\int_{0}^{2\pi }\int_{0}^{\pi }{\left(\sum_{m=1}^{M}\sum_{n=1}^{N}e^{j\left(\varphi_{I}{}_{m,n}+\varphi_{S}{}_{m,n}\right)}\right)}^{2}\sin\left(\theta \right)d\theta d\varphi }\right),$$

where *M* and *N* are the numbers of rows and columns of the array; θ and φ are the pitch angle and the yaw angle of observation point P, respectively; $\varphi_{I}{}_{m,n}$ and $\varphi_{S}{}_{m,n}$ are the in-array phase and the space phase of the T/R module at the position (*m*, *n*), respectively.

**Figure 7:**
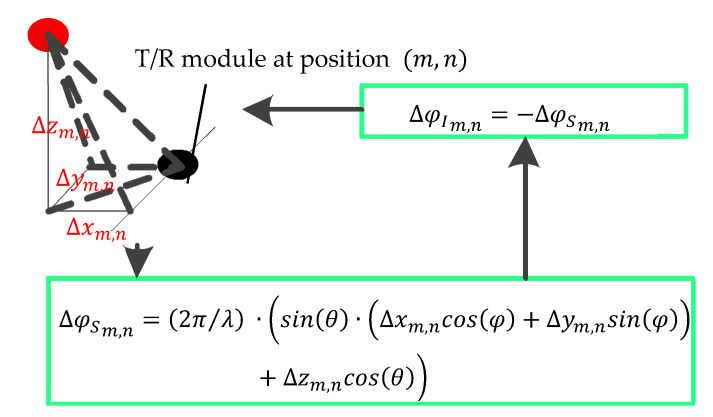
Principle of the thermal compensation control of the PAA. $∆\varphi_{S}{}_{m,n}$ is the change of the space phase, $∆\varphi_{I}{}_{m,n}$ is the adjusted value of the in-array phase, and $x_{m,n}$, $∆y_{m,n}$, and $∆z_{m,n}$ are the thermal deformations in the X, Y, and Z axes, respectively.

The phase of each T/R module includes the in-array phase and space phase. The in-array phase is controlled by a phase shifter. The space phase depends on the position of T/R modules on the panel. Then, if the space phase is changed by thermal deformation, the total phase can be corrected by adjusting the in-array phase, as shown in Figure 7. Equation (18) is the calculation method of the space phase:

(18) $$\varphi_{S}{}_{m,n}=\frac{2\pi }{\lambda }\;\left(\sin\left(\theta \right)\cdot \left(x_{m,n}cos\left(\varphi \right)+y_{m,n}sin\left(\varphi \right)\right)+z_{m,n}cos\left(\theta \right)\right)\;\;\;,$$

where θ and φ are the pitch angle and the yaw angle of observation point P, respectively; $x_{m,n}$,$\;y_{m,n}$, and $z_{m,n}$ are space coordinates of the T/R module at position (m,n); and λ is the wavelength.

### 4.2. Compensation Control Result

The thermal deformation measurement points were considered as T/R modules. Then, a 11×5 PAA was simulated by the simulation panel. In addition, the far-field pattern can be calculated by Equation (17). The wavelength (λ) was 31.25 mm (f=8 GHz, X band). The $\varphi_{I}{}_{m,n}$ values of all T/R modules were 0. Thus, the direction of observation point P was perpendicular to panel. Three far-field patterns of φ=0°,θ=−60°to60°were calculated as follows:

1. Ideal pattern: the T/R modules were in the ideal position;
2. Pattern after thermal deformation: the positions of the T/R modules were the ideal position + the thermal deformation measurement;
3. Pattern after compensation control: the positions of the T/R modules were the ideal position + the thermal deformation measurement. The in-array phase was adjusted for compensation control.

**Figure 8:**
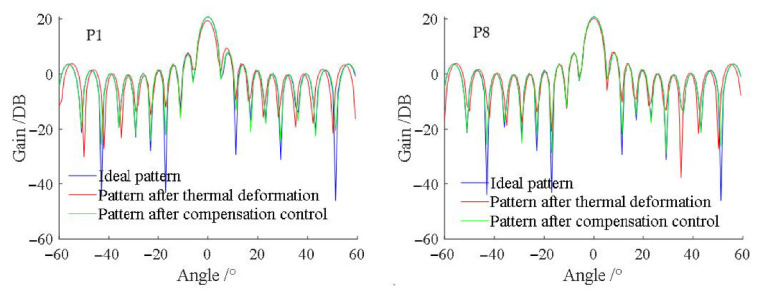
Patterns of the PAA (φ=0°,θ=−60° to 60°).

**Figure 9:**
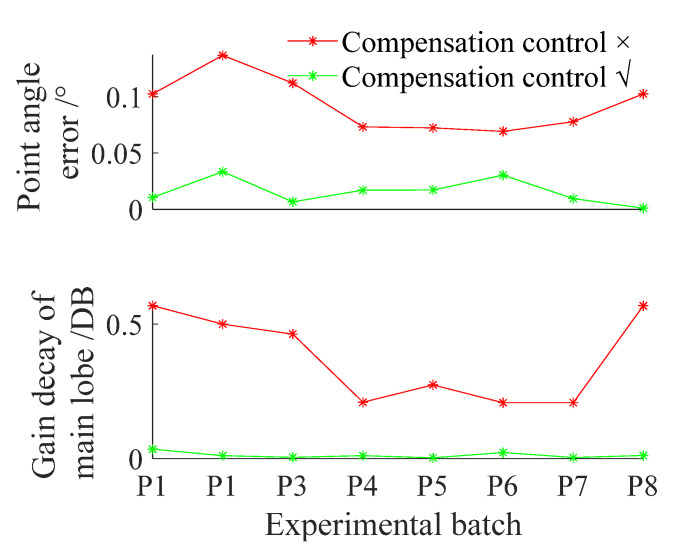
Compensation control effects.

The patterns of P1 and P8 are shown in Figure 8. The gain decays of the main lobe and the point angle errors of P1–P8 were calculated to measure the compensation control effect, as shown in Figure 9. In Figure 8 and Figure 9, after compensation control, the pattern was closer to the ideal one, and the gain decay of the main lobe and the point angle error were smaller.

## 5. Discussion

According to the simulation test results, the compensation control can correct the decay of the PAA performance caused by thermal deformation. However, it needs to accurately predict the thermal deformation of the panel of the PAA. However, factors such as the structural shape, assembly method, and material parameters will affect thermal deformation, and these parameters are difficult to measure because of the complex structure. Therefore, some assumptions and simplifications have to be made when using the traditional FEM to simulate thermal deformation, which leads to a decrease in the simulation accuracy. The CIPs-FEM algorithm can reproduce the thermal deformation law from the thermal deformation measurement data. The above factors and the effect of simplifications and assumptions on thermal deformation can be fitted by CIPs. Therefore, the thermal deformation model can be established on a simplified structure. In addition, the thermal deformation can be predicted on the same simplified structure. The experiment results showed the CIPs-FEM has a high accuracy of predicting thermal deformation. 

The most concerning issues for readers should be the robustness of the accuracy of the CIPs-FEM, which was analyzed as follows. The CIPs-FEM relies on data. It is like a memory algorithm that can extract and remember the thermal deformation characteristics of the structure from the temperature and thermal deformation data. Then, there are two reasons affecting the accuracy of the model:

1. The model problem: the thermal deformation characteristics extracted by the modeling algorithm are not real, that is, overfitting or underfitting.
2. The structural problem: the thermal deformation characteristics of the structure are not stable.

For the model problem, the nonuniform thermal deformation characteristics make the thermal deformation complicated. That is, after the same temperature change, the thermal deformations at different positions are different. In this study, the CIPs were introduced into each element separately for the FEM (as shown in Equation (4)). Then, each element could produce an independent anisotropic thermal normal strain and thermal shear strain. As long as the CIPs were appropriately selected, this modeling algorithm could fit complex thermal deformations. Therefore, the CIPs-FEM was not under-fitting. The experimental results can also prove this. For over-fitting, the experimental results showed that the CIPs-FEM model built by one batch of data had a good prediction effect for eight different temperature environments. Therefore, there was no obvious over-fitting. However, there is a potential collinearity problem that may lead to overfitting [25]. The collinearity problem will amplify the model’s sensitivity to errors in the modeling data, causing over-fitting. As a result, the model loses the prediction accuracy of the new data. We have encountered many overfitting problems in previous research, and biased regression can solve this problem well [26,27,28]. Ridge regression is a commonly used biased regression algorithm [29]. It only needs to add a bias term to Equation (13), as shown in Equation (19):

(19) $$\Omega_{T}={\left(\begin{array}{ccc} A_{1,1} & \cdots & A_{1,3\Lambda }\\ \vdots & \ddots & \vdots \\ A_{3\Lambda ,1} & \cdots & A_{3\Lambda ,3\Lambda } \end{array}+k\cdot {\left(\begin{array}{ccc} 1 & 0 & 0\\ 0 & \ddots & 0\\ 0 & 0 & 1 \end{array}\right)}_{3\Lambda \times 3\Lambda }\right)}^{-1}\left(\begin{array}{c} A_{1,0}\\ \vdots \\ A_{3\Lambda ,0} \end{array}\right)\;\;\;,$$

where k is the ridge parameter, and only a small ridge parameter is needed to solve the overfitting problem. However, the specific value needs further experimental research.

For the structural problem, the CIPs-FEM is a combination of the FEM and a data-driven model. It can only fit the thermal deformation characteristics in the measurement data. If the change in thermal deformation characteristics exceeds the measurement data, the prediction accuracy of the model will decrease. Obviously, the data-driven model cannot solve this problem. In this regard, the model can be rebuilt with new data. Alternatively, the thermal stability of the structure can be improved through a structural design. For example, the OKUMA company proposed a THERMO-FRIENDLY CONCEPT, to make the thermal deformation more stable when heated [30]. In addition, the proposed method converts thermal deformation into stress and strain. Therefore, if other factors that cause deformation, such as wind, the elastic deformation caused by the above factors can be directly superimposed and calculated by the FEM [31]. The specific approach needs to be studied in depth.

## 6. Conclusions

To solve the problem of thermal deformation modeling in the PAA thermal compensation control, a new thermal deformation modeling method combined with the FEM and a data-driven model was proposed in this study, referred to as the CIPs-FEM. Based on the FEM, new parameters called CIPs were introduced. CIPs can fit the influence of other factors on thermal deformation, such as the structural shape, assembly method, and material parameters. The CIPs can be obtained using the proposed IFEM algorithm with the measurement data of temperature and thermal deformation. Then, an accurate thermal deformation model can be built with simplified material parameters and structure shape, which significantly reduces the difficulty of the thermal deformation modeling. The experimental results showed that under different temperature environments, based on the same simplified conditions, the CIPs-FEM can increase the thermal deformation prediction accuracy of the PAA panel by 85.6% compared to the traditional FEM. The simulation results showed that the compensation control can effectively correct the decay of the PAA electrical performance caused by thermal deformation.

In addition to PAA thermal compensation control, the CIPs-FEM can also solve thermal deformation modeling problems in other fields, as long as there is a way to measure thermal deformation and temperature.

## Figures and Tables

**Table 1:** The voltages of the heaters.

Batch	Voltage (V)					
	R1	R2	R3	R4	R5	R6
M1	10	10	10	10	10	10
P1	20	20	20	20	20	20
P2	20	20	20	0	0	0
P3	20	0	0	20	20	0
P4	20	0	0	0	0	20
P5	0	20	0	20	0	20
P6	0	20	0	0	20	0
P7	0	0	20	20	0	0
P8	0	0	20	0	20	20

**Table 2:** Temperature measurement results.

Batch	Temperature (°C)			
	Row1	Row2	Row3	Row4
M1	14.8	14.2	13.9	14.5
P1	18.7	18.3	17.8	18.4
P2	14.2	12.9	11.6	9.6
P3	10.0	11.6	12.8	11.6
P4	8.3	9.0	8.7	9.2
P5	10.3	10.2	10.8	11.2
P6	8.9	9.1	9.0	9.0
P7	7.5	8.6	8.9	7.4
P8	10.0	10.1	11.3	12.7

## Data Availability

Not applicable.
